# Carbohydrate malabsorption in anorexia nervosa: a systematic review

**DOI:** 10.1186/s40337-022-00713-8

**Published:** 2022-12-06

**Authors:** Patrizia Buck, Jian Joli, Stephan Zipfel, Andreas Stengel

**Affiliations:** 1 Department of Psychosomatic Medicine and Psychotherapy, University Hospital Tübingen, Osianderstr. 5, 72076 Tübingen, Germany; 2 Charité Center for Internal Medicine and Dermatology, Medical Clinic for Psychosomatic Medicine, Charité‐Universitätsmedizin Berlin, Corporate Member of Freie Universität Berlin, Humboldt‐Universität zu Berlin, and Berlin Institute of Health, Berlin, Germany

**Keywords:** Anorexia nervosa, Carbohydrate malabsorption, Fructose malabsorption, Lactose intolerance

## Abstract

**Objectives:**

Anorexia nervosa (AN) is an eating disorder accompanied by a low body mass index and (self-) restricted food intake. Nutritional limitations can cause complaints of the digestive system, because of a disturbed absorption of food components. The absorption of carbohydrates may be seriously affected and reduced to a minimum. On this basis, a possible connection between AN, and the prevalence of gastrointestinal symptoms due to malabsorption was examined.

**Methods:**

For the systematic literature research with the aim of a better understanding of the topic the databases PubMed, Web of Science, Cochrane Library, Livivo and Google Scholar were used.

**Results:**

After the manual selection process of 2215 retrieved studies, 89 full texts were read and according to the predetermined eligibility criteria, finally 2 studies on the monosaccharide fructose and disaccharide lactose were included in this review.

**Conclusion:**

Malabsorption is often observed in patients with AN. It may contribute to the gastrointestinal complaints reported by patients and hamper body weight regain. Among others, mucosal atrophy and duodenal transporter dysfunction are discussed as main reasons. In the future more studies on carbohydrate malabsorption related to low body weight as observed in AN are warranted and may be conducted rather in an outpatient setting.

## Plain English summary

People with anorexia nervosa (AN) may experience a preference for foods containing fewer calories but more carbohydrates, e.g. fruits and vegetables. The consequences of this food restriction and selection may include malabsorption of sugars such as fructose and lactose, the mechanism of which is incompletely understood. This may contribute to symptoms similar to those seen in people with lactose intolerance, e.g. bloating, and make it harder for people to eat recommended foods. This paper presents a comprehensive literature search for research on this topic. However, only two studies were identified which highlights that further investigation is needed to explore this clinically relevant field.

## Introduction

Anorexia nervosa (AN) is a disease, which can be seen at any age but especially affects young female adolescents and adults [1]. It is characterized by an extremely restricted eating behavior or avoidance of eating, low body weight and fear of gaining weight. Further signs can be excessive workout (or other compensatory behavior) and secondary metabolic consequences caused by malnutrition [2, 3].

The metabolism and digestion can be severely impacted by AN which leads to gastrointestinal (GI) complaints like postprandial distress or distension of the abdomen and stomach due to slow gastric motility [4] which could—at least in part—further contribute to the low food intake of these patients [5]. In addition, functional GI disorders are frequent in patients with AN [6]. Lastly, subjects with AN may eat less of a regularly composed meal but tend to choose foods with low energy density, fewer calories, but large volume, for example fruits and vegetables or settle on a vegetarian diet with a higher amount of carbohydrates than protein or fat [7]. Reduction of foods containing high caloric carbohydrates like bread or cereals is common in patients with AN [8].

Nutrition, mental or physical stress as well as low body weight are factors which can affect the digestive system causing malabsorption of individual food components. Adverse food reactions (AFR) are often caused by carbohydrates, present in a healthy diet rich in fruits and greens. However, several carbohydrates including fructose and xylitol, just naming two, serve as sugar substitutes and are hidden in processed foods [9]. Ingestion of higher amounts of fructose may not be tolerated by patients with AN and cause GI symptoms [10]. The absorption of fructose is regulated by the apical glucose-5-transporter (GLUT5) and the basolateral glucose-2-transporter (GLUT2, a facultative transporter for glucose, fructose and galactose) in the small intestine. In the presence of glucose, GLUT2 can also be expressed on the apical epithelial membrane, thus facilitating fructose transport into the cell.

Potential effects on the impaired absorption capacity of fructose during exercise were mentioned in the studies of Fujisawa et al. [11] and Raithel et al. [9]. They have addressed acquired transport disorders of fructose after intensive physical training in healthy males in combination with a low-glucose diet and the interaction of the fructose transporter with other osmotically active substances like sugar substitutes [11]. Some parallels can be drawn to AN: Intensive physical training correlates with the consumption of fructose and can lead to incomplete intestinal absorption. They showed that a > 95% fructose solution induced a rapid increase in breath hydrogen during the training time in all 10 participants, which remained elevated during the following 2 h. Six of the individuals were tested at exercise and at rest. Malabsorption was observed in three of the six subjects at rest and in all subjects during training [11]. Fujisawa et al. further demonstrated that the simultaneous intake of fructose and glucose facilitates fructose absorption [11], which has been shown before [12–14]. This can be explained by the recruitment of the GLUT2 by glucose, which has been shown in animal studies [15, 16]. This recruitment of GLUT2 in humans has not been directly demonstrated but findings in humans with diabetes mellitus suggested higher GLUT2 mRNA in duodenal biopsy samples compared to healthy controls [17]. Lastly, a coupled transport via the disaccharidase-related transport system was hypothesized [18, 19]. Assuming a higher fructose consumption in patients with AN, all three mechanisms, GLUT5 [9], GLUT 2 and the coupled transport should be regarded. Despite the various transport possibilities, the quantity of absorbed fructose remains an individual parameter and may be more limited than previously expected [20].

The other important carbohydrate, lactose, can cause discomfort in the GI tract due to the genetic deficiency of the lactase enzyme [9], that could also be down-regulated [21] after longer-term avoidance of lactose [22] as seen in AN where the poor nutrient intake causes partial atrophy of villi in the small intestinal epithelium resulting in a reduced activity of lactase. Nichols et al. [21] compared the lactase activity in biopsy samples of 29 malnourished and 10 healthy infants. Before admission to the hospital the affected infants obtained a solution based on cow milk with added sucrose and starch in water, and after hospitalization they were nourished according to their age. The jejunal biopsy was taken 2 weeks after hospital admission. The analysis showed that the specific activity of lactase in infants with malnutrition was lower than in healthy controls depending on the level of villus atrophy. Similarly, the presence of lactase proteins was reduced in comparison to the control group [21]. Another consideration to possibly acquired lactose intolerance is the adaption following deprivation of lactose-containing products which was examined in the study of Cuatrecasas et al. [22]. Here, a decrease in lactose absorption in two subjects following 5 months of milk deprivation was shown. Furthermore, the whole (mixed-race) study group of 60 subjects classified as milk drinkers, intermediate drinkers or non-drinkers according to their self-reported amount of milk consumption showed the ability to absorb lactose in 20 of 23 (87%) drinkers, in 3 of 8 (37.5%) intermediate drinkers and in only 4 of 29 (13.8%) non-drinkers, why a correlation between lactase activity level and quantity of ingested lactose was hypothesized [22]. However, other studies [23, 24] could not verify this outcome, but Knudsen et al. [23] did not preclude the effect of lactose abstinence on lactase activity after longer duration of a diet (they only studied a period of 42 days), a condition likely given in patients with AN.

At first glance, studies examining malabsorption-associated GI complaints and symptoms after ingestion of common mono- and disaccharides in patients with AN seem rare. The current review examines the existing data on this topic in a systematic overview and attempts to describe possible mechanism(s) underlying these GI symptoms in patients with AN after consuming fructose or lactose, the two most common sugars contributing to carbohydrate malabsorption. Furthermore, possible malabsorption is discussed in the context of hampering weight regain therapy in patients with AN. Lastly, possibilities facilitating research in this field are discussed.

## Methods

### Registration

This systematic review was carried out according to the Preferred Reporting Items of Systematic Reviews and Meta-Analyses (PRISMA) criteria catalogue [25]. The protocol of the review has been registered in the International Prospective Register of Systematic Reviews (PROSPERO; CRD42022299295).

### Eligibility criteria

The research questions were developed according to the PICO or PECO scheme i.e. Population (P), Intervention (I) or rather Exposition (E), Comparison (C) und Outcome (O):

Population: Have patients with AN or a restrictive eating behavior…
Intervention/exposition: …determined by diagnostic tests like the hydrogen breath test…
Comparison: …in comparison to healthy individuals (where applicable)…
Outcome: …a malabsorption or GI complaints?

### Information sources and search strategy

For the literature search the databases PubMed, Web of Science, Cochrane Library und Livivo were used. The query took place on July 3rd 2021 with individual search strategies aligned to each database in order to identify all relevant articles on carbohydrate malabsorption associated with AN. Therefore, the most target-oriented components were selected from the PICO questionnaire, and in each case all possible expressions and synonyms were searched for and adapted to the keyword register of each database.

The following search terms and links were used: (1) PubMed: ("malabsorption syndromes"[MeSH Terms] OR "metabolic abnormalities"[Text Word] OR "carbohydrate metabolism"[Text Word] OR "carbohydrate malassimilation"[Text Word] OR malabsorption OR "carbohydrate maldigestion"[Text Word] OR "carbohydrate intolerance"[Text Word] OR "lactose intolerance"[Text Word] OR "lactose maldigestion"[Text Word] OR "fructose malabsorption"[Text Word]) AND (anorexia[MeSH Terms] OR "anore*"[Text Word] OR cachexia[MeSH Terms] OR "cache*"[Text Word] OR "cancer patients"[Text Word] OR "cancer cachexia"[Text Word]), (2) Web of Science: (TS = ("malabsorption syndromes") OR ALL = ("metabolic abnormalities") OR ALL = ("carbohydrate metabolism") OR ALL = ("carbohydrate malassimilation") OR ALL = (malabsorption) OR ALL = ("carbohydrate maldigestion") OR ALL = ("carbohydrate intolerance") OR TS = (lactose NEAR/2 intolerance) OR ALL = ("lactose maldigestion") OR ALL = ("fructose malabsorption")) AND (TS = (anorexia) OR ALL = (anore*) OR TS = (cachexia) OR ALL = (cache*) OR ALL = ("cancer patients") OR ALL = ("cancer cachexia")), (3) Cochrane Library: ((("Malabsorption Syndromes"[MeSH Terms]) OR ("metabolic abnormalities"[All Text] OR "carbohydrate metabolism"[All Text] OR "carbohydrate malassimilation"[All Text] OR “malabsorption” OR "carbohydrate maldigestion"[All Text] OR "carbohydrate intolerance"[All Text] OR (lactose near/2 intolerance)[All Text] OR "lactose maldigestion"[All Text] OR "fructose malabsorption"[All Text])) AND ((Anorexia[MeSH Terms]) OR (Cachexia[MeSH Terms]) OR (anore*[All Text] OR cache*[All Text] OR "cancer patients"[All Text] OR "cancer cachexia"[All Text]))), (4) Livivo: (MESH = ("malabsorption syndromes") OR FS = ("metabolic abnormalities" OR "carbohydrate metabolism" OR "carbohydrate malassimilation" OR (malabsorption) OR "carbohydrate maldigestion" OR "carbohydrate intolerance" OR "lactose intolerance" OR "lactose maldigestion" OR "fructose malabsorption")) AND (MESH = (anorexia OR cachexia) OR FS = ("anore*" OR "cache*" OR "cancer patients" OR "cancer cachexia")).

The hits were not limited regarding time of the studies, article type, year of publication and language. To achieve a search as comprehensive as possible, including gray literature, an additional query was made in Google Scholar, in which the first 200 hits were considered. The search term was: "carbohydrate malabsorption" AND (anorexia OR cachexia). An alert was also created in Web of Science to inform the first reviewer (PB) about new articles published after the query date that matched the search query.

### Selection process

The inclusion and exclusion criteria for literature screening were based on the PICOS or PECOS scheme but were adapted and specified for the selection of relevant studies. The categories population, methods (corresponding to intervention or exposure), clinical picture (corresponding to outcome), and study characteristics (corresponding to study design) were employed. The order of priority with which each category was considered was: (1) population, (2) study characteristics, (3) disease pattern and, where possible, (4) methods.

Population: Studies included had to be performed in patients with AN with all subtypes (restrictive or purging type), or weight-reducing diseases with restrictive eating behavior. There was no restriction regarding age or gender.

Study characteristics: Studies were considered as possibly eligible if they contained data from one or more patients and encompassed clinical trials, case-control studies and case series. Non-original studies (meeting/conference/congress abstracts, notes, and narrative reviews), animal studies, articles with non-topic-specific content, editorials, dissertations, books or letters were excluded from further examination. Reviews, except narrative reviews, were not generally excluded directly, but rather examined for potentially important primary sources if relevant to the topic.

Clinical picture: Studies reporting malabsorption of commonly occurring mono- or disaccharides (lactose, fructose, glucose) or non-immunologically induced carbohydrate intolerance were included. The origin of malabsorption was also considered. If it was due to bacterial malabsorption due to antibiotic therapy, GI resection, chemotherapy-induced intolerance, secondary to diseases such as Crohn's disease, genetic metabolic or immunological diseases (e.g., celiac disease, hereditary fructose intolerance), studies were excluded.

Methods: Based on the respective GI complaints of the patients, only few diagnostic tests were considered. These included lactose or fructose H_2_ exhalation breath tests as previously described as gold standard for the diagnosis of lactose intolerance or fructose malabsorption [10, 26]. Studies involving H_2_ breath tests with xylose or lactulose or measurements after intravenous glucose and non-oral glucose were excluded. Likewise, studies could not be included if the oxidation rate (O_2_ uptake and CO_2_ release) of glucose was tested, because this was outside of the score of the current review.

Articles which met the criteria described above and written in English were eligible for inclusion.

### Data collection process

**Fig. 1:**
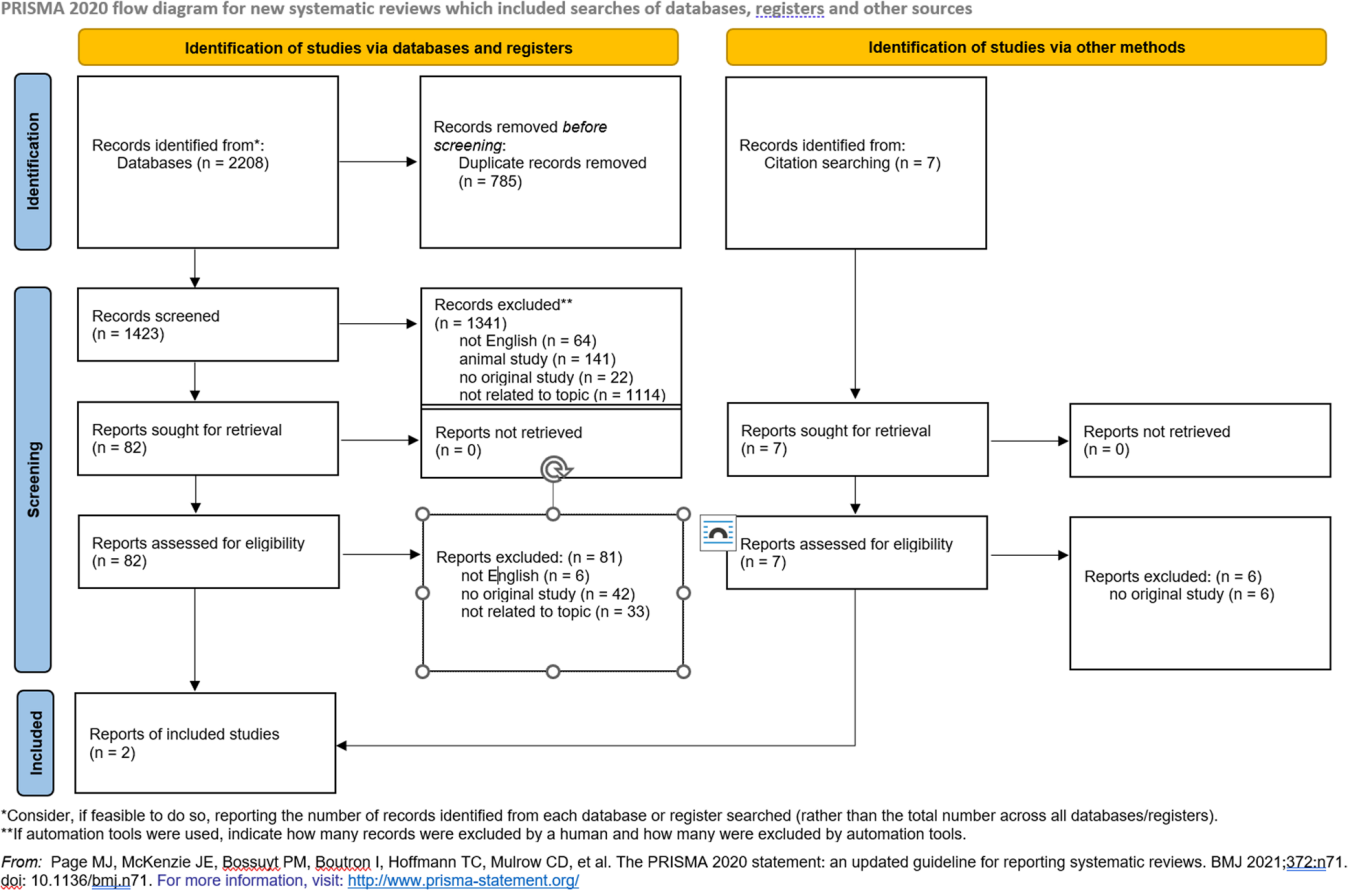
Prisma flow chart

After the database search, first the duplicates were removed. This step was performed independently by two investigators (PB and JJ) and the number of titles was compared afterwards. This was followed by the independent screening by the two authors considering title and abstract. Next, hits were matched and classified as suitable, and in case of discrepancies, the investigators discussed the articles in question. This process left 82 articles for full-text screening, which the two authors also screened in-depth independently. In the case of a review, they each checked the articles' references for possible matching sources, of which the abstracts were again read individually and, after deciding on suitability, the full texts of these (7 articles) were also read. Eight studies were identified as possibly suited for the current review. Both agreed on 87.5% (7 out of 8 studies). After consultation with a third investigator (AS), further 6 studies were excluded due to inappropriate patient group or data collection. Of the articles found by alert in Web of Science, none could be included because they did not meet the inclusion criteria. Thus, two studies were included in the current review for data extraction and discussion. The entire selection process is shown in the PRISMA flowchart (Fig. 1).

### Extracted data

The following data were extracted from both studies included in the review: study type, existing disease (AN) as well as duration of disease until data collection, number and gender of the subjects participating in the study, intervention or exposure performed, whether a control group was present, the observation period as well as the applied measurement methods and results concluded from the data collected (Table 1).

**Table 1:** Studies included in the review

References	Study type	Entity	Number of participants	Intervention/exposition	Control	Observation period/parameters and tests	Key results
Friesen et al. [27]	Case-control study	Eating disorders (EDs)	26 female patients with ED: anorexia nervosa (n = 10), bulimia nervosa (n = 5), ED not otherwise specified (n = 11: 6 purging type, 5 restricting type)	50 g glucose solution and 25 g fructose-5 g sorbitol (F–S) solution	Healthy normal weight women (n = 20)	2 consecutive days/gastrointestinal (GI) symptoms: abdominal pain and discomfort, bloating, abdominal distension, burping, nausea, increased bowel movement, feeling of fullness, borborygmi (determined before and hourly following ingestion over a period of 3 h), hydrogen breath concentration (in ppm)	GI symptoms in patients with ED especially with a low BMI (≤ 17,5 kg/m^2^) were greater following F–S (n = 15)—including 8 with AN, 13 defined as malabsorbers based on the hydrogen breath concentration (≥ 20 ppm)—than following glucose (n = 1) 80% of patients with AN exhibited a symptom response (total symptom score of 5 or greater) to F–S No difference in the peak levels of the hydrogen breath concentration between ED malabsorbers and healthy control malabsorbers No significant difference in symptom scores between ED malabsorbers and absorbers GI transit time in ED malabsorbers longer than in control malabsorbers following F–S
Täljemark et al. [28]	Cross-sectional study	Restrictive eating problems (REP), mental and GI comorbidities	Boys and girls with REP (n = 95) including 3 with the diagnosis AN, selected from 9- or 12-year old Swedish twin children (n = 19,130)	None	Children without REP (n = 18,261)	Data from telephone interviews with parents of 9- or 12-year old Swedish twins out of the sub-study CATSS 9/12 (Child and adolescent twin study in Sweden) in 2004, birth cohorts between 1992 and 2000 were considered, to determine mental and physical health problems in children including questions about previous clinically diagnosed ED, REP definition based on the eating module in the Autism, Tics-AD/HD and other Disorders (A-TAC) inventory, Mann–Whitney U tests to examine the differences in the mean number of coexisting disorders among boys and girls	Most prevalent coexisting GI problems in children with REP were constipation (n = 21, 22.1% of children with REP), lactose intolerance (n = 12, 12.6% of children with REP) and food allergy (n = 11, 11.6% of children with REP) The odds ratio (OR) for coexisting lactose intolerance in girls was 2.91, most prevalent coexisting psychiatric problems were attention deficit and hyperactivity disorder (ADHD) and learning disorder (LD, both n = 34 or 35.8% of children with REP)

### Risk of bias in studies

The two included studies were assessed for methodological quality or potential bias. The assessment was based on published Critical Appraisal Tools, which were modified, where it seemed necessary:

Case control study: Critical Appraisal Skills Program (CASP) Case Control Study Checklist.

Cross-sectional study: Joanna Briggs Institute (JBI) Checklist for Analytical Cross-Sectional Studies.

On the basis of the Critical Appraisal Tool Checklists, the studies were checked for their risks of bias categorized in ‘low risk’ (green dot), ‘some concerns’ (yellow dot) and ‘high risk’ (red dot). Therefore, the reviewers PB and JJ evaluated the studies on five various bias forms: selection of study participants, whether deviations from the intended performance existed, missing outcome data, measurement of intervention and selection of results. The assessment of the investigators is shown in Table 2. Although the second article listed in Table 2 is at high risk of bias, it was not excluded in order to contrast the two common sugars, fructose and lactose, and to provide a comparison between subjective (self-reported) and objective (measured) malabsorption.

**Table 2:**
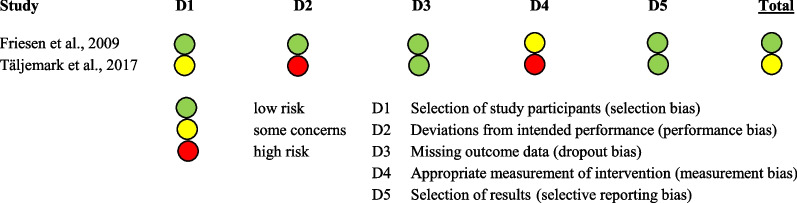
Assessment of risks of bias

## Results

### Summary of study characteristics

In both studies, patients had low body weight but different definitions of eating disorders (EDs).

#### Case control study with ED and GI symptoms

The article by Friesen et al. was published in 2009 [27]. The EDs were classified as AN in 10, bulimia nervosa (BN) in 5 and EDs not otherwise specified (EDNOS, including purging and restricting type) in 11 inpatient women. A few of the tested patients had a GI disease which failed to comply with the category clinical picture in the selection process, but the existing disease did not affect the presence or absence of GI symptoms after substrate administration. The participants in the control group were all female and of normal weight. The substrate testing in ED patients was conducted on two consecutive days and took place 2 weeks after their hospital admission.

#### Cross sectional study with ED and GI symptoms

The article by Täljemark et al. was published in 2017 [28]. The ED focused on was called restrictive eating problems (REP), a newly defined diagnose since most of the children and young adolescents, displaying disordered eating behavior, did not meet the criteria for classical EDs, but displayed an increased risk to develop an ED like AN later. REP was diagnosed by asking parents of 9- or 12-year-old children during a telephone interview: (1) Questions were derived from validated questionnaires—the eating module in the Autism, Tics-AD/HD and other disorders (A-TAC) inventory—e.g. if the children failed to gain enough weight for 1 year or had fear of gaining weight. (2) Questions on clinically diagnosed EDs (AN, BN) in the past. Of the selected 95 children with REP, three had an existing diagnosis of AN.

The children participating in the study were selected from the sub-study CATSS 9/12 (Child and Adolescent Twin Study in Sweden) which started in 2004. A control group of 18,261 children without REP was used to examine the prevalence of coexisting psychiatric or GI problems.

#### Identification of EDs

A major difference between the two included studies was the identification of existing EDs. In the study of Friesen et al. [27] all female patients had to have an ED diagnosis given by a psychiatrist and psychologist, whereas the study of Täljemark et al. [28] considered the assessment of the interviewed parents about their children’s food intake behavior.

### Summary of study results

#### Study outcomes for case control study with ED and GI symptoms

**Fig. 2:**
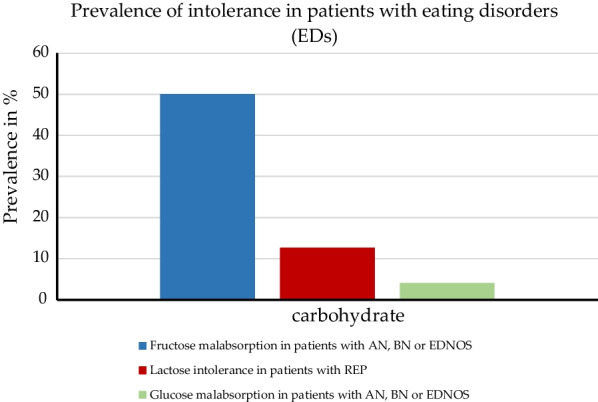
Prevalence of lactose intolerance (self-reported), glucose and fructose malabsorption (measured) in patients with eating disorders or restrictive eating problems. Abbreviations: AN, anorexia nervosa; BN, bulimia nervosa; ED, eating disorders; EDNOS, eating disorders not otherwise specified; REP, restrictive eating problems

Subjective assessment of GI symptoms: The female patients with ED were asked hourly over a period of 3 h after ingestion of the fructose/sorbitol (F–S) solution about their type of complaint and its severity. A symptom score for each complaint determined at every hour and for the whole test duration of 3 h after ingestion of the solution was used. Only 1 of 20 (5%) healthy controls complained of one or more GI symptoms, whereas 55% of patients with EDs had, after summation of all symptom scores, in total a symptom score of five or higher (scale ranging according to the degree of each symptom from 0 [absent] to 3 [severe]) 3 h after F–S provocation. Thereof, eight (57%) patients had a diagnosis of AN, one (7%) of BN and five (36%) of EDNOS. Only in one (4%) patient with ED a symptom response to glucose was observed (Fig. 2). The accumulated symptom scores for patients with a BMI ≤ 17.5 kg/m^2^ were greater than in patients with a higher BMI [27].

Objective assessment of GI symptoms: The H_2_ breath test as a diagnostic method was used to identify a possible relationship between GI complaints and incomplete small bowel (mal)absorption of the ingested F–S solution. The test results were used to categorize the patients with ED and healthy women into malabsorbers—in case of a breath hydrogen level ≥ 20 ppm above baseline—or absorbers. The H_2_ breath measurement (irrespective of symptoms) showed a result different from the subjective outcomes: hydrogen breath levels indicated a F–S malabsorption in 13 of the 26 (50%) female patients and also in 14 of the 20 (70%) healthy controls (Fig. 2). The hydrogen breath peak levels between malabsorbers in the ED group did not differ from those in the healthy control group.

It is to note that patients with ED and malabsorption showed a longer mouth to cecum transit time (106 ± 35 min) than control subjects (without ED) and those malabsorption alone (54 ± 25 min). No difference was observed in total symptom scores between ED malabsorbers (8 ± 7) and ED absorbers (5 ± 3) after provocation with F–S.

#### Study outcomes for cross sectional study with ED and GI symptoms

Subjective assessment of GI symptoms was based on the answers of the parents of twin children with REP. The telephone interview questions regarding GI conditions like lactose intolerance or other food or nutritional allergies had the answer options yes or no. Out of the 95 children (40 boys and 55 girls), 12 (12.6%, 5 male and 7 female) were reported to have lactose intolerance (Fig. 2) and 11 (11.6%, 5 male and 6 female) food allergies. A difference between boys and girls regarding REP and coexisting GI problems was determined: Girls with REP had a twofold risk of lactose intolerance compared to boys. In comparison to 18,261 healthy children without REP the rate of coexisting GI conditions was higher, because in the control group only 984 (5.4%), among them 535 (5.7%) boys and 449 (5%) girls, had a lactose intolerance and 1,532 (8.4%), including 811 (8.7%) boys and 721 (8.1%) girls, had a food allergy.

The most frequent symptoms mentioned in both studies after ingestion of fructose or due to lactose intolerance were abdominal pain, nausea, bloating and flatulence [27], but were not investigated in particular in the study on children with REP [28]. Furthermore, complaints like discomfort, distension of the abdomen, belching, loose stool or increased frequency of bowel motions, sensation of fullness and borborygmi were assessed only in one study [27].

### Results of assessment of risk of bias

The studies were checked on their possible bias by means of the underlying design and characteristics. An overview is given in Table 2. Both included studies, a case-control study and cross-sectional study, had a great observational share because of the questionnaires used to assess complaints. That is why the measurement bias was at middle (some concerns) to high risk. This method was also the basis of the participant selection in one study; hence it was classified as a middle risk of bias. The cross-sectional study compared the prevalence of complaints between affected and non-affected children but did not state how the data of the control group were compiled, which caused a high performance bias. The rate of drop out bias due to missing outcome data was at low risk in both studies, as well as the selective reporting bias.

## Discussion

As shown in the current review, there is little data available on carbohydrate malabsorption in patients with AN, with only two studies identified after an extensive systematic search.

In the two analyzed studies GI complaints (abdominal pain, nausea, bloating and flatulence) in patients with EDs were reported related to the ingestion of fructose(-sorbitol) or lactose [27, 28]. Aside from constipation as a frequently reported symptom in patients with AN [29], lactose intolerance was likewise named as the most prevalent coexisting GI condition occurring in young girls with restricted eating behavior [28]. The study by Friesen et al. [27] described an increase of the above-mentioned complaints in patients with a BMI ≤ 17.5 kg/m^2^, therefore more common in AN, after ingestion of a fructose-sorbitol solution. The impaired absorption of fructose-sorbitol in patients with AN may be due to the similar chemical structure of sorbitol and fructose [30] or the metabolic process in which sorbitol can be converted into fructose and therefore overloading the GLUT5 resulting in worsened fructose transport capacity [9]. Sorbitol, a sugar alcohol, is, beside fructose, especially used in low energy products [10, 27] which are preferentially chosen by anorexic patients [31]. However, disturbed transporters or enzymes [9, 11, 20, 21] do not necessarily result in a reduced absorption capacity of carbohydrates. Other causes need to be considered, e.g. the perturbation of the GI microbiota in AN compared to normal-weight subjects [32, 33]. A shifted bacteria abundance towards potentially pathogenic bacterial genera was described [32], whereas a reduced abundance of carbohydrate utilizing taxa could be observed [33]. This is potentially reversible as after weight gain, there was almost no difference for the carbohydrate utilizing *Roseburia spp* between patients with AN and normal-weight participants [33]. Lastly, low body weight itself has already been linked to malabsorption as the study by D’Costa [34] showed. However, they used xylose in participants with severe weight loss due to diabetic neuropathic cachexia. A delayed absorption of xylose could be determined, and based on this carbohydrate malabsorption was suggested. Overall, it still remains unclear as to what extent underweight and specifically AN and malabsorption of carbohydrates connect with each other.

During our literature search, we had to observe several limitations: First, the two studies included in this review were heterogenous with regards to their participants, methods, and study designs, therefore making it impossible to give an exact statement about the prevalence of fructose malabsorption or lactose intolerance in patients with AN or to comment on a possible improvement of GI complaints after normalization of eating behavior and/or weight recovery. Another limitation was the underrepresentation of male patients since men are considerably less frequently affected by EDs [35]. Finally, the lack of research in this topic could be explained by the difficulties in conducting studies with these patients: the recruitment of patients with AN suffering from psychological and physical complications within the context of the ED and their participation in a study, which deals with the intake of carbohydrates and additionally bearing the risk of increased GI symptoms and thus further causing deterioration of the already impaired general well-being, is impeded. Beyond that, repeated measurements within the same subjects over the course of the treatment would be desired but is also hampered by the points mentioned above and will likely result in a considerable dropout rate. Therefore, future studies with larger samples are likely to consist predominantly of patients with milder forms of AN from the outpatient setting, and thus severe forms of AN may be underrepresented. Necessary measuring devices and expertise required for carrying out the H_2_ testing as the gold standard to examine carbohydrate malabsorption, however, should not pose an impediment because the needed equipment (for detecting the hydrogen content in the exhalation air as an indicator for malabsorption) and materials (specific amount of carbohydrates dissolved in water) [10] are affordable as well as feasible.

In summary, data on carbohydrate malabsorption in patients with EDs, especially AN, is sparse. However, identification of the prevalence of carbohydrate malabsorption, using hydrogen breath testing as a non-invasive and inexpensive tool [10], in AN is of clinical importance. GI symptoms are frequently reported by patients with EDs and can greatly complicate therapy, especially in the case of AN [36]. Understanding the underlying mechanisms of GI complaints in patients with AN triggered by impaired carbohydrate absorption will help to modify weight regain therapy and possibly improve the therapeutic outcome.

## Data Availability

Not applicable.

## Abbreviations

AFR: Adverse food reactions
AN: Anorexia nervosa
BN: Bulimia nervosa
CH: Carbohydrate
ED: Eating disorder
EDNOS: Eating disorders not otherwise specified
F–S: Fructose/sorbitol
GI: Gastrointestinal
REP: Restrictive eating problems
